# Structures and mechanism of chitin synthase and its inhibition by antifungal drug Nikkomycin Z

**DOI:** 10.1038/s41421-022-00495-y

**Published:** 2022-12-06

**Authors:** Yanan Wu, Min Zhang, Yizheng Yang, Xuyang Ding, Ping Yang, Kai Huang, Xinlin Hu, Mingjie Zhang, Xiaotian Liu, Hongjun Yu

**Affiliations:** 1 Department of Biochemistry and Molecular Biology, School of Basic Medicine, Tongji Medical College, Huazhong University of Science and Technology, Wuhan, Hubei China; 2 Department of Pathogen Biology, School of Basic Medicine, Tongji Medical College, Huazhong University of Science and Technology, Wuhan, Hubei China; 3 Department of Cardiology, Union Hospital, Tongji Medical College, Huazhong University of Science and Technology, Wuhan, Hubei China; 4 Clinical Center of Human Gene Research, Union Hospital, Tongji Medical College, Huazhong University of Science and Technology, Wuhan, Hubei China; 5 School of Life Sciences, Southern University of Science and Technology, Shenzhen, Guangdong China; 6 Greater Bay Biomedical Innocenter, Shenzhen Bay Laboratory, Shenzhen, Guangdong China; 7 Cell Architecture Research Center, Huazhong University of Science and Technology, Wuhan, Hubei China

**Keywords:** Electron microscopy, Molecular biology

Dear Editor,

Chitin, one of the most common polysaccharides in nature, is produced by fungi, insects, fish, etc. Chitin is a linear N-acetylglucosamine (GlcNAc) polymer with β-1,4-linkage and is synthesized by membrane-integrated chitin synthase (Chs). As the essential polysaccharide in cell walls of prominent fungal pathogens, chitin plays vital roles in cell wall assembly and cell integrity maintenance for fungi^1^. Chitin biosynthesis, therefore, becomes an appealing target for antifungal drug development^1,2^. One well-known example is Nikkomycin Z (NikZ), the competitive inhibitor of Chs^3,4^ (Fig. 1a). NikZ is under clinical development for treating coccidioidomycosis, blastomycosis, and histoplasmosis^2,5,6^. However, the mechanism of action of Chs and its inhibition by NikZ remain enigmatic.

**Fig. 1:**
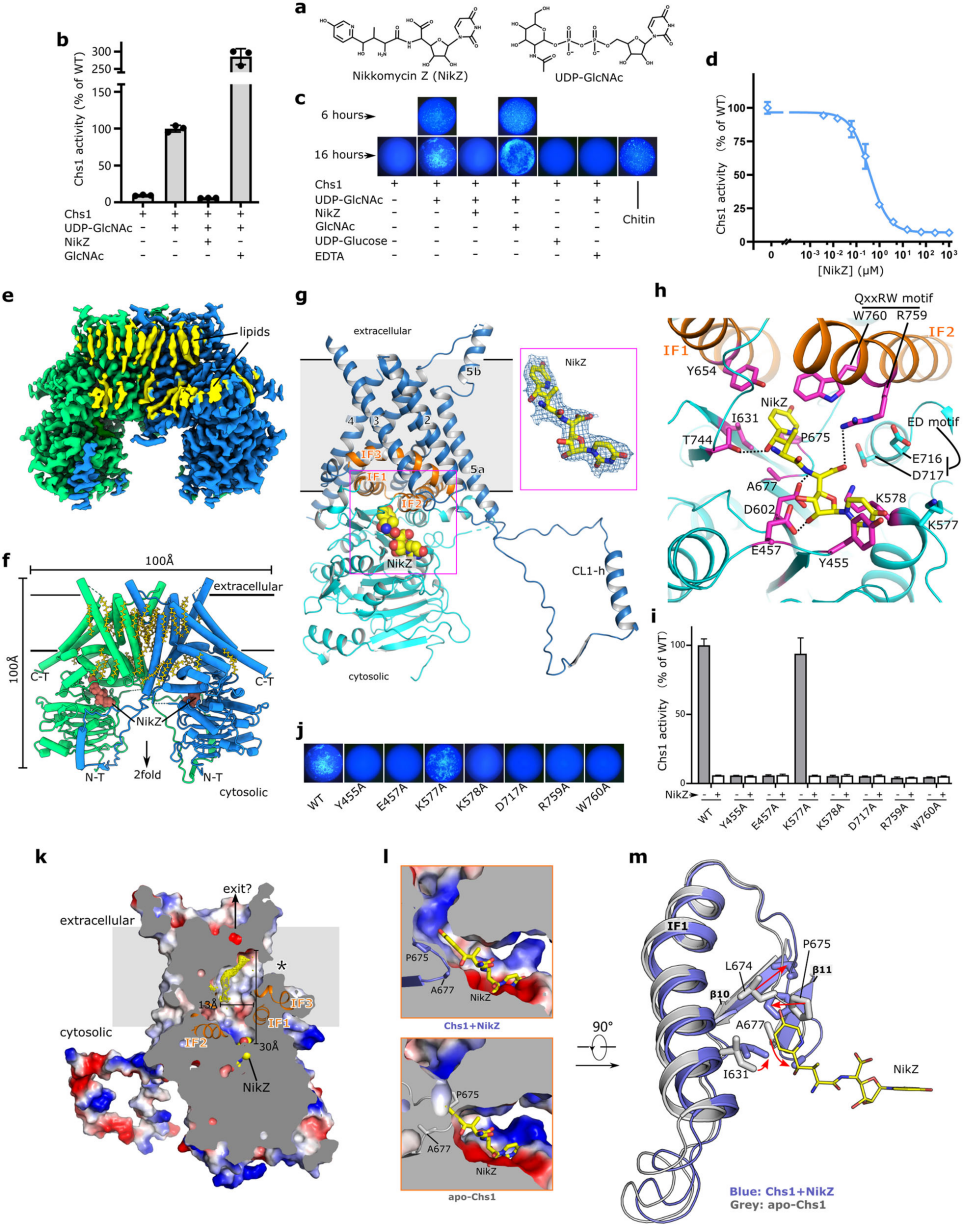
Cryo-EM structures of *S. cerevisiae* Chs1 in apo-form and in complex with antifungal drug NikZ. **a** Chemical structures of Chs1 substrate UDP-GlcNAc and antifungal drug NikZ. **b** Activity assay of Chs1. **c** The reactants under different reaction conditions and times (6 h, 16 h) were stained by calcofluor white to visualize chitin formed. Commercial chitin was included as a positive control. **d** Inhibition profile of Chs1 activities by NikZ. **e** Cryo-EM map of Chs1 complexed with NikZ. Two protomers are colored in green and blue, respectively, and lipid densities in yellow. **f** Dimeric overall structure of Chs1/NikZ complex. Lipids are shown as yellow sticks and NikZ as red spheres. **g** Cartoon representation of one Chs1 protomer with bound NikZ (as yellow sphere). Inset, NikZ (as yellow stick) superimposed with its corresponding density (as blue mesh). **h** Coordination of NikZ in Chs1 active site. NikZ-interacting residues are shown as magenta sticks. Black dashed lines indicate polar interactions. E716 and D717 (cyan sticks) from the conserved ‘ED motif’, are close to but not contacting NikZ. **i** In vitro activities of Chs1 active site mutants with or without NikZ. **j** The reactants (reaction time: 16 h) in the presence of Chs1 active site mutants were stained by calcofluor white to visualized chitin formed. **k** The transmembrane domain encloses a membrane tunnel for chitin translocation. A lipid-like density (yellow mesh) resides within the membrane tunnel. The arrow indicates a possible exit for translocation. The asterisk indicates a membrane-wrapped lateral opening to the membrane tunnel. **l** Structural comparison of their active sites between the Chs1/NikZ complex and apo-Chs1. A ‘plug loop’ (in cartoon with two residing residues P675 and A677 shown in sticks) exhibits altered conformations to block (apo-state) or open (NikZ-bound state) the path connecting the active site and the membrane tunnel. **m** Conformational changes in Chs1 active site upon NikZ binding. The red arrows indicate the altered conformations of ‘plug loop’ and I631 on IF1 before and after drug binding. All assays were performed in triplicates. The error bars indicate SD.

Fungal species typically contain multiple members of Chs. In *S. cerevisiae*, Chs activities have been ascribed to Chs1–3. We expressed *S. cerevisiae* Chs1 and purified it as a dimer (Supplementary Fig. S1a, b). The purified Chs1 is active and can synthesize chitin in vitro, exhibiting donor specificity toward UDP-GlcNAc (Fig. 1b, c). Moreover, Chs1 activity can be significantly stimulated by GlcNAc and abolished by EDTA, consistent with the previous study^7^. Notably, NikZ can inhibit Chs1 activity, with IC_50_ of 0.367 μM (Fig. 1b–d), confirming Chs1 as the target of NikZ^3,4^. We then determined the cryo-EM structure of Chs1 in complex with NikZ, to a resolution of 2.9 Å with an imposed C2 symmetry (Fig. 1e; Supplementary Figs. S1, S2 and Table S1). Two NikZ molecules, 40 lipid alkyl chains and 2 phospholipids were identified in the structure. Unsolved regions include N-terminal residues (1–377) and three short loop segments (residues 699–703, 897–907, 1076–1084).

The Chs1 dimer measures ~100 Å × 100 Å × 50 Å. Each Chs1 protomer contains an N-terminal cytoplasmic domain (residues 378–783) and a C-terminal transmembrane domain (residues 784–1131) (Supplementary Fig. S3a, b). The cytoplasmic domain adopts a GT-A fold characteristic of glycosyltransferases^8^. The transmembrane domain contains six TM helices (TM1–6), one interfacial helix IF3 and one long cytosolic loop (CL1; residues 969–1027) between TM4 and TM5. CL1 is partially structured and contains a helix (CL1-h). It extends ~50 Å toward the cytoplasmic domain of the other protomer (Fig. 1f). Notably, the membrane–cytoplasm interface features three interfacial helices (IF1–3) lying almost parallel to the membrane surface. Of them, IF1–2 are from the cytoplasmic domain and IF3 is from the transmembrane domain (Supplementary Fig. S3).

Regarding the dimer assembly, two Chs1 protomers form extensive interactions (Fig. 1f; Supplementary Fig. S4a). Firstly, the dimer shows a domain-swapped configuration, as the α-helix (CL1-h) in the extended CL1 contacts the cytoplasmic domain of the opposing protomer (Supplementary Fig. S4b). Secondly, the dimerization interface is predominantly mediated by extensive van der Waals interactions between TM2 in one protomer and TM5 in the neighboring protomer (Supplementary Fig. S4d, f). At this interface, three lipid alkyl chains were observed (L1–L3), securing the contacts between TM2 and TM5 (Supplementary Fig. S4e, f). Thirdly, on the cytosolic side, L854 on TM2 of each protomer contacts the other to stabilize the dimer (Supplementary Fig. S4c). The K857 and N856 on the loop immediately following TM2 contact two residues from the opposite protomer (W969 on CL1 and N1038 on TM5a) through van der Waals packing and hydrogen bond, respectively. Lastly, along the 2-fold symmetry axis, two symmetry-related phospholipids (PL1, PL2) were observed in the extracellular membrane leaflet (Supplementary Fig. S4d, g). They locate within a sizable membrane chamber. This chamber is enclosed by TM2 and TM5 from two protomers and is further sealed by the interaction of I1087–I1087 from two protomers. These two phospholipids are closely packed and form extensive interactions with surrounding residues, strengthening the dimer architecture of Chs1 (Supplementary Fig. S4g, h).

NikZ binds to a cytosolic tunnel near the membrane–water interface (Fig. 1g). It makes contact with multiple conserved Chs1 residues (Fig. 1h; Supplementary Figs. S5–S7). Specifically, the uracil group is sandwiched between K578 and Y455 and the ribose group is engaged with polar interaction with E457 (Supplementary Fig. S5a, b). The pyridin-3-ol group packs with surrounding I631, Y654, P675, A677, and W760, which define a narrowed path leading into the membrane domain (Supplementary Fig. S5c). Three pairs of polar contacts are also formed: R759 with the carboxyl group near the ribose group, D602 with the amino group of the central peptide linkage, T744 with the hydroxyl group near the pyridin-3-ol group (Supplementary Fig. S5a, b).

Further analysis suggests key Chs1 residues in catalysis or substrate binding. Of the drug-interacting residues, R759 and W760 are part of the ‘QxxRW motif’ conserved in GT2 family (Fig. 1h), which has been suggested to participate in cellulose and hyaluronan synthesis^9,10^. We also noted that ‘ED motif’, another conserved motif proposed for catalysis in GT2 family^9^, locates near the carboxyl group of NikZ (Fig. 1h). Given that NikZ contains the uridine group as UDP-GlcNAc (Fig. 1a), we deduce a model for Chs1 catalysis. In this model, Y455 and K578 stack with the uracil group of the donor; E457 interacts with donor ribose through hydrogen bonds; D717 of the ‘ED motif’ serves as a general catalytic base; R759 of the ‘QxxRW’ motif may coordinate the diphosphate of the donor while W760 of ‘QxxRW motif’ may coordinate acceptor substrate. Comparison of the active sites between Chs1 and cellulose synthase BcsA shows that these key residues are all conserved (Supplementary Fig. S8). Individual substitution of these residues (Y455, E457, K578, D717, R759, or W760) with alanines all markedly reduced the enzymatic activity (Fig. 1i, j). In contrast, alanine substitution of K577, a residue close to but not involved in donor binding, had no obvious effects on the enzyme activity. Notably, the crucial residues identified above are well conserved in chitin synthases from multiple fungal pathogens, indicating the conserved catalytic mechanism (Supplementary Figs. S6, S7).

The Chs1 structure also suggests a potential membrane path for chitin translocation. Chs1 structure features a hollow interior within its membrane domain (Fig. 1k). A membrane tunnel is enclosed by TM1, TM3–4, TM6, and IF1–3 (Supplementary Fig. S9a, b). This membrane tunnel locates above the cytosolic tunnel holding the active site, with IF1–3 defining the narrowed path between them. NikZ bound in the active site has its pyridin-3-ol group inserted into this membrane tunnel (Fig. 1k). The pyridine-3-ol group packs with W760 in IF2, a residue guarding the path to the membrane tunnel (Fig. 1h; Supplementary Fig. S2d). Therefore, the membrane tunnel likely defines the passage for chitin translocation. This membrane tunnel is predominantly hydrophobic (Fig. 1k), which is lined mainly with hydrophobic residues (Supplementary Fig. S9d, e). Consistently, a lipid-like density is observed near the upper end of this tunnel (Fig. 1k). These observations emphasize the hydrophobic nature of the tunnel. Notably, the membrane tunnel of Chs1 has a lateral opening between TM3 and TM4 (Fig. 1k). This opening is buried deeply in detergent micelle and likewise buried in a lipid bilayer in the native plasma membrane, seemingly precluding its role in chitin release. Instead, the upper end of the membrane tunnel is pretty close to the extracellular space where the tunnel converges at the intersection among TM1, TM3–4, and TM6 (Supplementary Fig. S9a–c). This indicates a potential gate for chitin release, though conformational changes would have to occur.

We further determined the structure of apo-Chs1 (Supplementary Fig. S10). Comparing it with Chs/NikZ structure reveals no overall conformational differences (Supplementary Fig. S10g). Surprisingly, we noted that the path connecting the cytosolic tunnel of the active site and the membrane tunnel for translocation is blocked in the apo-Chs1 (Fig. 1l). This is caused by re-arrangements of a loop (residues 668–677) between IF1 and β11 upon NikZ binding (Fig. 1m; Supplementary Fig. S10h, i). Specifically, I631 has its side chain rotated ~90°. P675 and A677 flip in opposite directions, respectively. L674 flips upside down and move away from NikZ. These changes occur near W760 from IF2, together leading to the formation of a tunnel to accommodate the pyridine-3-ol group of NikZ (Fig. 1h). This opens the path between the active site and the membrane tunnel for translocation (Fig. 1l). Therefore, we named the loop with altered conformations the ‘plug loop’, which may serve as a cytoplasmic gate of the transmembrane channel and prevent the leakage of non-substrate molecules.

In summary, our structures of *S. cerevisiae* Chs1 (in apo-form and in NikZ-bound form) advance mechanistic understanding of fungal cell wall chitin biosynthesis, revealing key catalytic residues and the chitin translocation path within the membrane. Our work provides new insights into the GT2 family of membrane-integrated GTs and highlights the Chs oligomer in chitin fibrillogenesis (see Supplementary Discussions). Our study indicates that NikZ competes with the substrate and blocks the path for chitin translocation, supporting NikZ as a competitive inhibitor of Chs1^3,4^. These insights provide a foundation for the development of new antifungal drugs.

## Supplementary information

Supplementary information

## Data Availability

EM density maps have been deposited in the EMDB under the accession codes EMD-33422 and EMD-33423. Atomic coordinates have been deposited in the PDB under the accession codes 7XS6 and 7XS7.

## References

[1] Cortes, J. C. G., Curto, M. A., Carvalho, V. S. D., Perez, P. & Ribas, J. C. *Biotechnol. Adv*. **37**, 107352 (2019). 10.1016/j.biotechadv.2019.02.008 30797093

[2] Perfect, J. R. *Nat. Rev. Drug Discov*. **16**, 603–616 (2017). 10.1038/nrd.2017.46 PMC5760994 28496146

[3] Gaughran, J. P., Lai, M. H., Kirsch, D. R. & Silverman, S. J. *J. Bacteriol*. **176**, 5857–5860 (1994). 10.1128/jb.176.18.5857-5860.1994 PMC196793 8083179

[4] Cabib, E. *Antimicrob. Agents Chemother*. **35**, 170–173 (1991). 10.1128/aac.35.1.170 PMC244960 2014972

[5] Sass, G. et al. *Antimicrob. Agents Chemother*. **65**, e0028521 (2021). 10.1128/AAC.00285-21 PMC8448119 34252303

[6] Goldberg, J. et al. *Antimicrob. Agents Chemother*. **44**, 1624–1629 (2000). 10.1128/aac.44.6.1624-1629.2000 PMC89923 10817719

[7] Sburlati, A. & Cabib, E. *J. Biol. Chem*. **261**, 15147–15152 (1986). 2945823

[8] Lairson, L. L., Henrissat, B., Davies, G. J. & Withers, S. G. *Annu. Rev. Biochem*. **77**, 521–555 (2008). 10.1146/annurev.biochem.76.061005.092322 18518825

[9] Morgan, J. L., Strumillo, J. & Zimmer, J. *Nature* **493**, 181–186 (2013). 10.1038/nature11744 PMC3542415 23222542

[10] Maloney, F. P. et al. *Nature* **604**, 195–201 (2022). 10.1038/s41586-022-04534-2 PMC9358715 35355017

