# Supplementary material for: Structures and mechanism of chitin synthase and its inhibition by antifungal drug Nikkomycin Z
Source: Cell Discov. 2022 Dec 6;8:129. doi: 10.1038/s41421-022-00495-y (PMC9726829; doi:10.1038/s41421-022-00495-y)
Supplement: Supplementary file 1 — Supplementary information [file 41421_2022_495_MOESM1_ESM.pdf]

Supplementary Materials for

Structures and mechanism of chitin synthase and its inhibition by antifungal drug Nikkomycin Z

Yanan Wu[1,2]#, Min Zhang[1,2]#, Yizheng Yang[1]#, Xuyang Ding[1], Ping Yang[1], Kai Huang[3,4], Xinlin Hu[1], Mingjie Zhang[5,6], Xiaotian Liu[5]*, Hongjun Yu[1,7]*.

Affiliations:

[1]Department of Biochemistry and Molecular Biology, School of Basic Medicine, Tongji Medical College, Huazhong University of Science and Technology, Wuhan, China.

[2]Department of Pathogen Biology, School of Basic Medicine, Tongji Medical College, Huazhong University of Science and Technology, Wuhan, China.

[3]Department of Cardiology, Union Hospital, Tongji Medical College, Huazhong University of Science and Technology, Wuhan, China.

[4]Clinical Center of Human Gene Research, Union Hospital, Tongji Medical College, Huazhong University of Science and Technology, Wuhan, China.

[5]School of Life Sciences, Southern University of Science and Technology, Shenzhen, Guangdong, China.

[6]Greater Bay Biomedical Innocenter, Shenzhen Bay Laboratory, Shenzhen, 518036, China.

[7]Cell Architecture Research Center, Huazhong University of Science and Technology, Wuhan, China.

#These authors contributed equally to this work

*Corresponding authors. Emails: liuxt@sustech.edu.cn or hongjun_yu@hust.edu.cn

This file includes:

Supplementary Discussions

Materials and Methods

Additional references

Supplementary Figs. S1 to S11

Supplementary Table S1

Supplementary Discussions

Our Chs1 structure provides new insights into the GT2 family of membrane-integrated processive GTs (previously structurally represented by cellulose synthases such as BcsA and CesA). We found that Chs1, the GT2 member, adopts a novel fold. The structural comparison between Chs1 and BcsA reveals their limited overall structural similarity, with resemblance mainly limited to their cytosolic GT domains (**Fig. S11a**). Within GT domain and at the membrane-cytoplasm interface, their characteristic motifs such as the 'ED motif', the 'QxxRW motif', the interfacial helix IF1-3 are well matched between the two structures, suggesting a conserved catalytic mechanism in synthesizing chitin and cellulose (**Fig. S11b, c**). In contrast, the transmembrane domains of Chs1 and BcsA vary dramatically in numbers of TM helices as well as the topologies (**Fig. S11d, e**), resulting in differently organized transmembrane tunnels above the active site and likely different modes of product translocations through membranes.

Our study reveals that Chs1 forms a compact dimer through the extensive contacts on membrane domain as well as through a domain-swapping of the cytosolic CL1 region. This dimerization mode is consistent with that of the recently reported structure of *Candida albicans* Chs2 where they show quite similar conformations (with root-mean-square-deviation RMSD of 1.2-Å over 666 aligned residues)[1]. Consistently, oligomer was also observed during the purification of insect chitin synthase[2]. Therefore, it is conceivable that the oligomerization of chitin synthase could underlie the formation of chitin microfibrils (**Supplementary Fig. S9c**). There are two basic polymorphic types of chitin microfibrils: α-form and β-form where individual chitin chains are organized in anti-parallel and parallel manner, respectively. Though this complexity leaves the assembly mechanism enigmatic, it would be tempting to speculate on the basis of dimeric architecture of Chs. For β-chitin, the two polymer chains extended on individual protomer of Chs dimer may directly assemble in a parallel manner. For α-chitin, the polymer synthesized on one Chs protomer could fold on itself through intra-chain contacts to form a sheet with anti-parallel arrangement [3]; this could be followed by the inter-chain packing of a similar sheet from the other Chs protomer, forming the network of chitin microfibrils [4].

Materials and Methods

Protein expression and purification

The codon-optimized full-length cDNA of *S. cerevisiae* Chs1 (Uniprot ID P08004) was subcloned into pCAG vector with a C-terminal 3xFLAG tag. HEK 293F suspension cells (Invitrogen) were cultured in freestyle 293 medium at 37 °C under 5% CO_2 in shaker. The cultured cell at a density of ~ 2×10^6 cells per milliliter were transiently transfected with expression plasmid and polyethylenimines (PEI). For 1-liter cell culture, approximately 1.4 mg of plasmid was pre-mixed with 6 mg of PEI in 50 ml of fresh medium for 15-30 minutes at room temperature before transfection. The transfected cells were cultured for 48 hours before harvest.

For protein purification, the harvested cells were lysed by sonication on ice in buffer: 25 mM HEPES pH 7.4, 150 mM NaCl, 2 mM $MgCl_2$ and protease inhibitor cocktail (Roche). Then, the membrane was solved with 1% (w/v) n-Dodecyl-β-D-Maltopyranoside (DDM, Anatrace) and 0.1% cholesteryl hemisuccinate Tris salt (CHS, Anatrace) at 4 °C for 2 hours. After centrifugation at 20,000 g for 1 hour, the collected supernatant was incubated with anti-Flag M2 affinity resin (Sigma). The resin was washed with washing buffer 25 mM HEPES pH 7.4, 150 mM NaCl, 2 mM $MgCl_2$ and 0.04% glyco-diosgenin (GDN, Anatrace) and eluted with washing buffer supplemented with 200 μg/ml 3×FLAG peptide. The eluate was further purified with gel filtration chromatography (Superose 6 10/300 GL column, Citiva) in buffer: 25 mM HEPES pH 7.4, 150 mM NaCl, 2 mM $MgCl_2$ and 0.04% glyco-diosgenin (GDN, Anatrace). The peak fractions were pooled and concentrated for biochemical studies or cryo-EM grid preparation.

Grid preparation and Cryo-EM data acquisition

For cryo-EM analysis, three-microliter aliquots of the purified Chs1 at the concentration of 5 mg/ml were applied to glow-discharged grids (Quantfoil R1.2/1.3 Au, 300 mesh). Then the grids were blotted for 5 seconds with 100% humidity at 4 °C and were plunge-frozen in liquid ethane using an Vitrobot Mark IV system (Thermo Fisher Scientific). For Nikkomycin Z (NikZ) treated sample, NikZ at final concentration of 1 mM was added to purified Chs1 (5 mg/ml). The mixture was incubated at 4 °C for 30 minutes before freezing the cryo-EM grids. Cryo-EM data were recorded on a K3 camera (Gatan) in a 300 kV FEI Titan Krios electron microscopy (FEI). The datasets were automatedly collected with EPU

(FEI). Micrographs were recorded in counting mode at a nominal magnification of 130,000× with a physical pixel size of 0.92 Å. Defocus values varied from -1.0 μm to -3 μm. A total exposure of 1.3 seconds was dose-fractionated into 32 sub-frames, resulting in a total accumulated dose of 50 electrons per $Å^2$.

Cryo-EM data processing

The recorded movies were motion-corrected using MotionCor2 [5]. CTFFIND4 was then used to estimated Contrast transfer function (CTF) parameter for individual micrograph [6]. Further data processing was carried out using RELION-3.0 [7] and similar strategy was used for the dataset of apo-Chs1 and the dataset of Chs1 treated with Nikkoymcin Z. For each dataset, a set of ~1000 particles were manually picked, which were subjected to Relion 2D classification to generate the templates for autopicking. Initial 3D model was generated using Relion 3D initial model module. One or two rounds of 3D classification without the application of symmetry were performed to eliminate particles of poor quality. Subsequently, the good particles were selected for one round of 3D classification with C2 symmetry. The 3D class with high-resolution structural features were combined for 3D refinement, polishing and postprocess. Finally, for apo-Chs1 dataset, a map at an overall resolution of 3.2 Å was obtained from 93641 particles; for Chs1+Nikkomycin Z dataset, a map at an overall resolution of 2.9 Å was obtained from 188337 particles. The resolution was estimated using the gold standard Fourier shell correlation 0.143 criterion [8]. The distribution of local resolution distribution was estimated using ResMap [9].

Model building and refinement

The initial model of Chs1 was obtained from the map of Chs1 complexed with Nikkomycin Z, generated de novo with the map_to_model in PHENIX [10]. It was further improved by manual adjustments and rebuilding in COOT [11]. The building process was aided by the good structural features around secondary structures, transmembrane helices as well as bulky residues. Nikkomycin Z and bound lipids were added according their densities. The refinement of Chs1+Nikkomycin Z model was carried out using real_space_refine module in PHENIX [10]. To build the apo-Chs1 model, the initial model was obtained from the Chs1+Nikkomycin Z model. It was rigid-body docked into the apo-Chs1 map and was further manually inspected and adjusted in COOT [11]. Then the

apo-Chs1 model was refined using PHENIX [10]. The final models were assessed using MOLPROBITY [12]. Chimera, ChimeraX and PyMOL (Schrödinger, LLC.) were used to prepare the figures [13,14]. Statistics of the 3D reconstruction and model refinement were provided in **Supplementary Table S1**. EM density maps have been deposited in the Electron Microscopy Data Bank (EMDB) with accession codes EMD-33422 and EMD-33423. Atomic coordinates have been deposited in the Protein Data Bank (PDB) with accession codes PDB 7XS6 and 7XS7.

Chs1 activity assay

Site-directed mutagenesis was done with PCR overlapping extension and confirmed by sequencing. The Chs1 variants was purified as described above. The Chs1 activity was measured by monitoring the UDP generated, using UDP-Glo Glycosyltransferase Assay kit (Promega). Chs1 variants (2 μg) was added to 20 μL reaction mixture in total containing 50 mM Tris-HCl pH 7.4, 150 mM NaCl, 2 mM $MgCl_2$ and 0.04% GDN. The reaction was initiated by adding UDP-GlcNAc to a final concentration of 4 mM. Reactions were carried out at 30 °C for 1 hour. Luminescence was recorded using a Pherastar FS system (BMG Labtech). In the case of inhibitor profiling, serial dilutions of Nikkomycin Z drug were first incubated with Chs1 for 5 minutes at room temperature before adding other components to react.

Calcofluor White Staining

The synthesized chitin polymer was visualized by the fluorescent dye Calcofluor White [15]. Wild-type or mutant Chs1 samples (2 μg) were added to a 20 μL reaction mixture prepared as described above. Reactions were carried out at 30 °C for 16 hours. Then the reactants were incubated with equal volume of Calcofluor White Stain solution (Sigma) in the dark for 15 minutes. A two-microliter aliquot of the reaction mixture was taken onto a slide and observed under a fluorescence microscope (MSX2-C, Mshot) with an excitation wavelength of 365 nm and an emission wavelength of 433 nm.

Additional References

1 Ren, Z. *et al.* Structural basis for inhibition and regulation of a chitin synthase from Candida albicans. *Nature structural & molecular biology* **29**, 653-664, doi:10.1038/s41594-022-00791-x (2022).

2 Maue, L., Meissner, D. & Merzendorfer, H. Purification of an active, oligomeric chitin synthase complex from the midgut of the tobacco hornworm. *Insect biochemistry and molecular biology* **39**, 654-659, doi:10.1016/j.ibmb.2009.06.005 (2009).

3 Lenardon, M. D., Munro, C. A. & Gow, N. A. Chitin synthesis and fungal pathogenesis. *Curr Opin Microbiol* **13**, 416-423, doi:10.1016/j.mib.2010.05.002 (2010).

4 Sikorski, P., Hori, R. & Wada, M. Revisit of alpha-Chitin Crystal Structure Using High Resolution X-ray Diffraction Data. *Biomacromolecules* **10**, 1100-1105, doi:10.1021/bm801251e (2009).

5 Zheng, S. Q. *et al.* MotionCor2: anisotropic correction of beam-induced motion for improved cryo-electron microscopy. *Nature methods* **14**, 331-332, doi:10.1038/nmeth.4193 (2017).

6 Rohou, A. & Grigorieff, N. CTFFIND4: Fast and accurate defocus estimation from electron micrographs. *Journal of structural biology* **192**, 216-221, doi:10.1016/j.jsb.2015.08.008 (2015).

7 Zivanov, J. *et al.* New tools for automated high-resolution cryo-EM structure determination in RELION-3. *eLife* **7**, doi:10.7554/eLife.42166 (2018).

8 Rosenthal, P. B. & Henderson, R. Optimal determination of particle orientation, absolute hand, and contrast loss in single-particle electron cryomicroscopy. *Journal of molecular biology* **333**, 721-745, doi:10.1016/j.jmb.2003.07.013 (2003).

9 Kucukelbir, A., Sigworth, F. J. & Tagare, H. D. Quantifying the local resolution of cryo-EM density maps. *Nature methods* **11**, 63-65, doi:10.1038/nmeth.2727 (2014).

10 Adams, P. D. *et al.* PHENIX: a comprehensive Python-based system for macromolecular structure solution. *Acta crystallographica. Section D, Biological crystallography* **66**, 213-221, doi:10.1107/S0907444909052925 (2010).

11 Emsley, P., Lohkamp, B., Scott, W. G. & Cowtan, K. Features and development of Coot. *Acta crystallographica. Section D, Biological crystallography* **66**, 486-501, doi:10.1107/S0907444910007493 (2010).

12 Chen, V. B. *et al.* MolProbity: all-atom structure validation for macromolecular crystallography. *Acta crystallographica. Section D, Biological crystallography* **66**, 12-21, doi:10.1107/S0907444909042073 (2010).

13 Pettersen, E. F. *et al.* UCSF chimera - A visualization system for exploratory research and analysis. *J Comput Chem* **25**, 1605-1612, doi:10.1002/jcc.20084 (2004).

14 Pettersen, E. F. *et al.* UCSF ChimeraX: Structure visualization for researchers, educators, and developers. *Protein science : a publication of the Protein Society* **30**, 70-82, doi:10.1002/pro.3943 (2021).

15 Elorza, M. V., Rico, H. & Sentandreu, R. Calcofluor white alters the assembly of chitin fibrils in Saccharomyces cerevisiae and Candida albicans cells. *J Gen Microbiol* **129**, 1577-1582, doi:10.1099/00221287-129-5-1577 (1983).

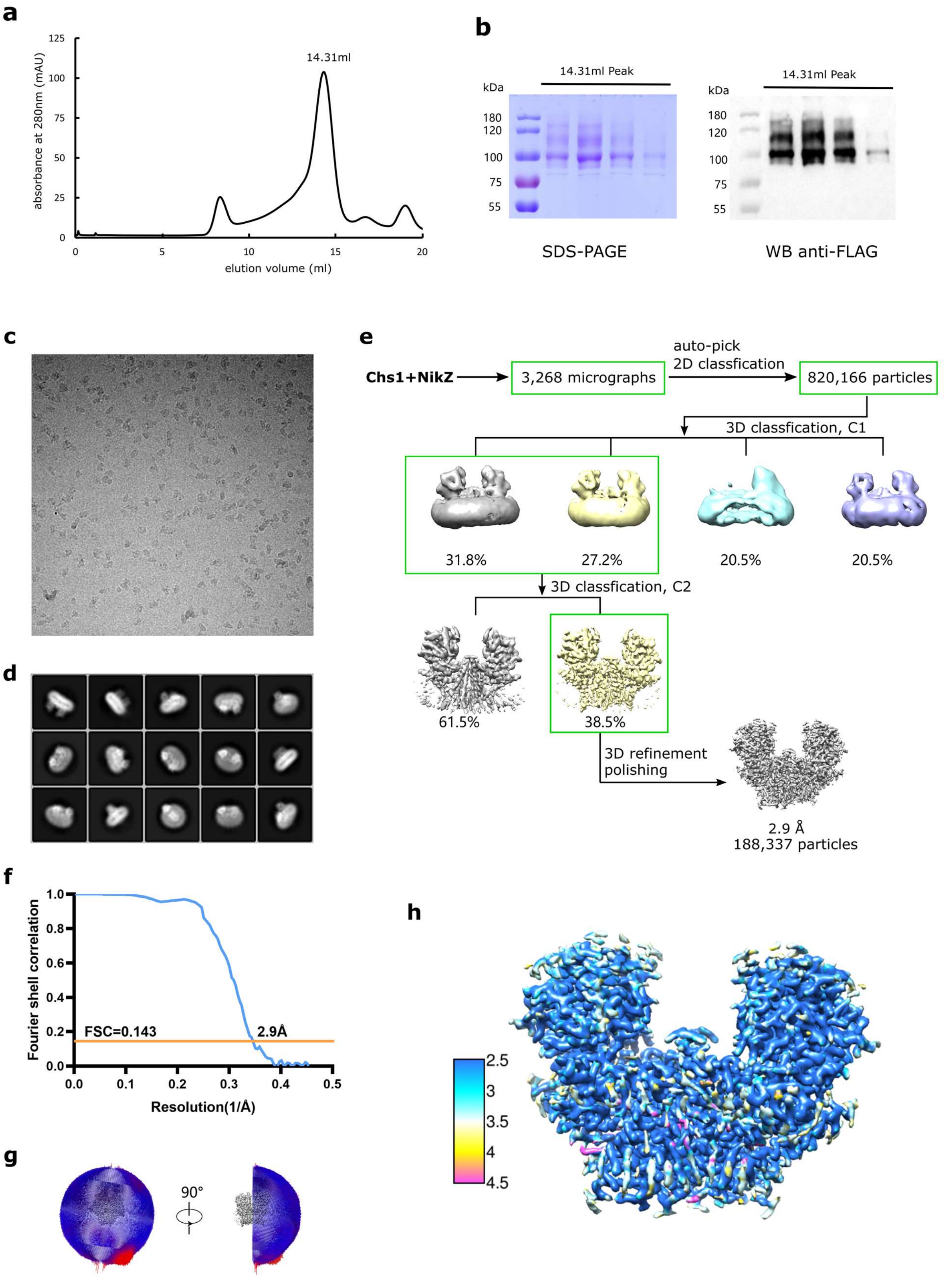

Supplementary Fig. S1. Sample preparation and CryoEM analysis of *S. cerevisiae* Chs1 in complex with Nikkomycin Z.

a, Gel filtration chromatography profile of Chs1.

b, SDS-PAGE (left) and western blot (right) analysis of the fractions of the monodisperse elution peak in (**a**). The Chs1 suffered from proteolysis during purification and the major population approximates ~100 kDa, consistent with the existence of a long (~300 residues) N-terminal unstructured region within the total length of 1131 residues.

c, Representative cryo-EM micrograph of Chs1 complexed with Nikkomycin Z.

d, Representative 2D class averages.

e, Flowchart for cryo-EM data processing.

f, Gold-standard Fourier shell correlation (FSC) curve.

g, Cutaway views of the angular distribution of all particles used in the final 3D reconstruction.

h, Local resolution distribution of the final cryo-EM map, calculated with ResMap.

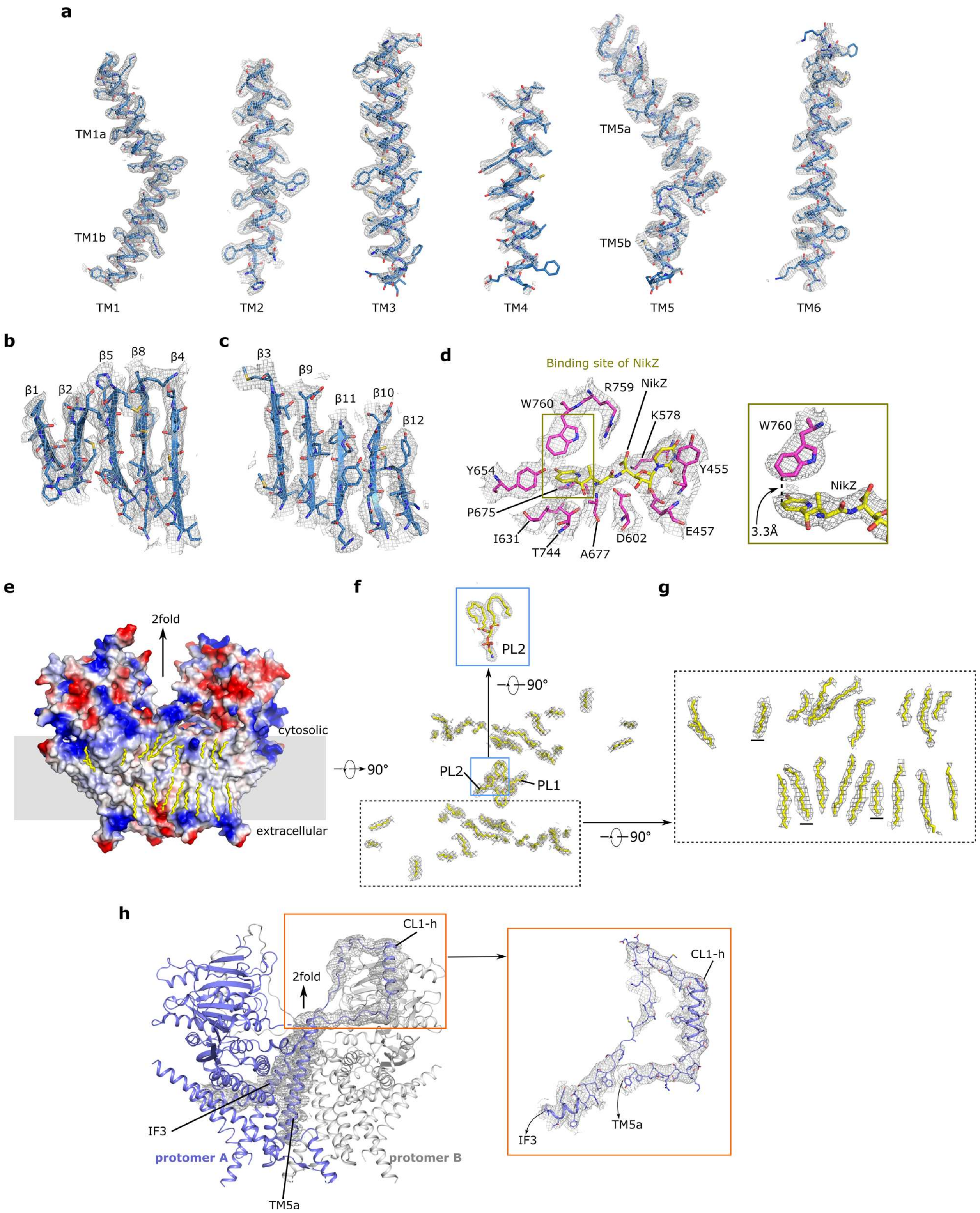

Supplementary Fig. S2. Fit of cryo-EM map with the model of Chs1 bound to Nikkomycin Z at representative regions.

a, Cryo-EM densities of transmembrane helices TM1-7.

b-c, Cryo-EM densities of 10 beta-strands of the central beta sheet in GT domain.

d, Cryo-EM densities corresponding to the bound Nikkomycin Z and its interacting residues. Inset, close-up view of the boxed region (brown line) in (**d**) shows the stacking between W760 and pyridine-3-ol group. The closest packing distance (3.3 Å) between these two groups is shown as the dashed line.

e, Electrostatic surface representations of the Chs1 dimer with bound lipids (yellow sticks).

f, Cryo-EM densities of the bound lipids in Chs1 dimer structure. These include two phospholipids (PL1 and PL2) involved in Chs1 dimerization (see **Supplementary Fig. S4g-h** for more details). Inset, close-up view of one (PL2) of the two phospholipids.

g, Detailed view of the cryo-EM densities corresponding to lipids at one membrane side of Chs1 dimer. Three densities underlined are blobby densities tentatively assigned as lipid alkyl chains.

h, Unsharpened map for the entire domain-swapped CL1 region as well as its preceding region IF3 and following region TM5a. For clearness, the density is only shown for one Chs1 protomer. Inset, cryo-EM densities of the entire CL1 region. For clearness, only parts of the connecting regions (IF3 and TM5a as labelled) are shown.

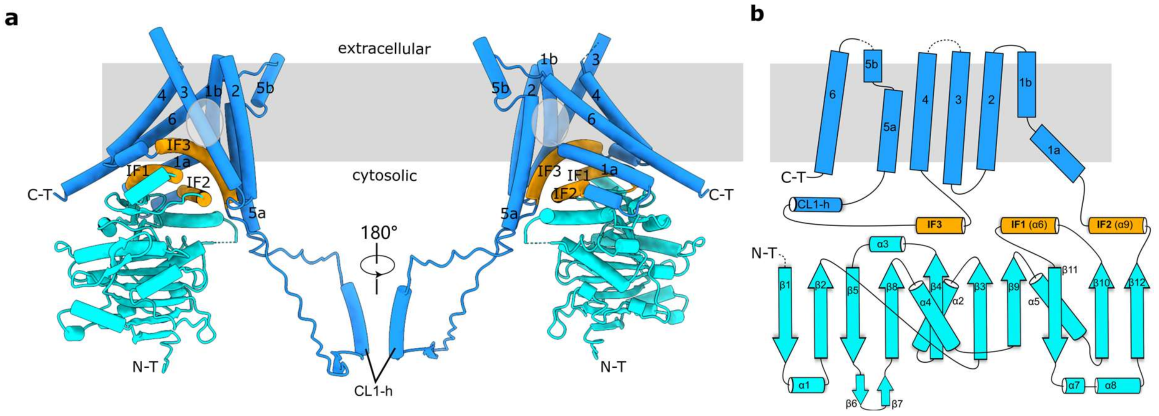

Supplementary Fig. S3. Overall structure of one Chs1 protomer.

a, Structure of one Chs1 protomer, colored by structural elements: TM helices (denoted by numbers) in blue; cytoplasmic domain in cyan; interfacial helices (IF1-3) in orange. The grey oval marks the position of a tunnel formed within transmembrane domain implicated in chitin translocation.

b, Topology diagram of Chs1 protomer. The cytoplasmic domain adopts a GT-A fold where ten beta-strands (β1-5, 8-12) form the central beta-sheet, which is surrounded by multiple alpha-helices.

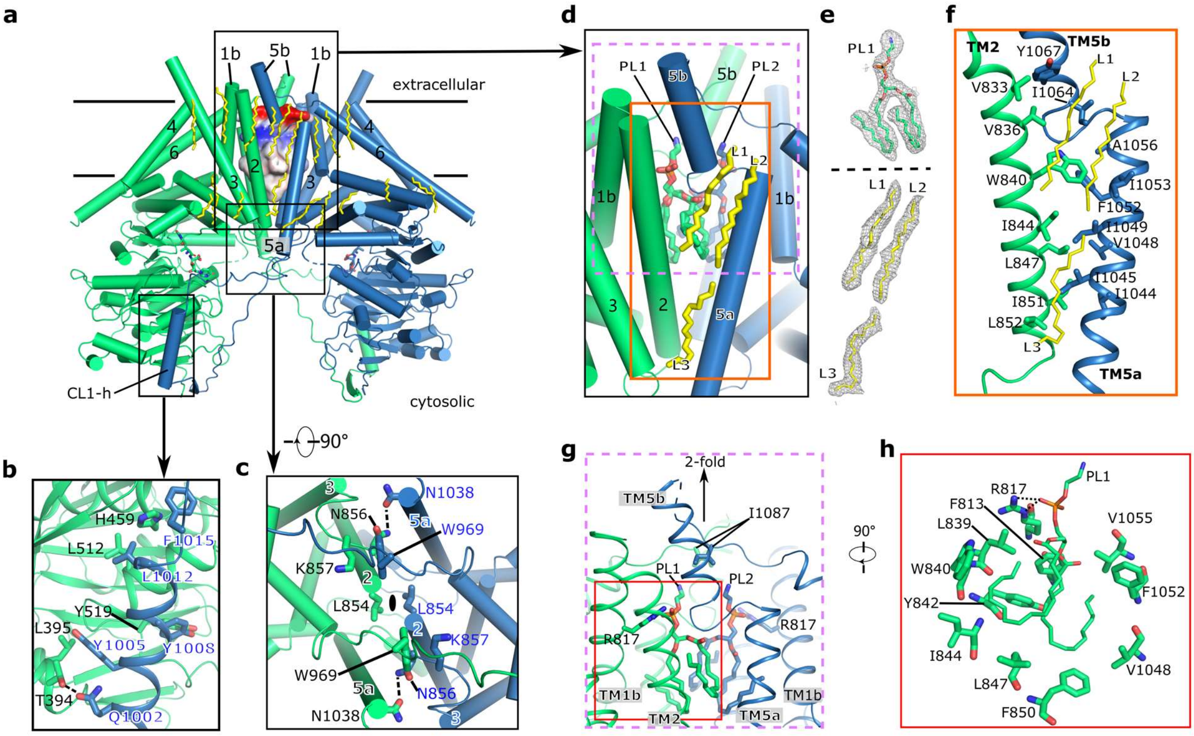

Supplementary Fig. S4. Assembly of Chs1 dimer.

a, The structure of Chs1 dimer is viewed parallel to the membrane. Two protomers are colored in green and blue, respectively. Transmembrane helices are denoted by numbers. A sizable membrane chamber located between two protomers is shown as electrostatic surface representation (internal). Ordered lipids are shown in yellow sticks.

b, Detailed view of the domain-swapping region marked by box in (**a**). Residues mediating dimerization are shown as sticks. This interface involves one pair of polar contact between Q1002 and T394 and involves van de Waals contacts among hydrophobic residues Y1005, Y1008, L1012 and F1015 of one protomer and L395, Y519, L512 and H459 of the other protomer.

c, Detailed view of the dimerization interface at the cytosolic side as marked by box in (**a**).

d, Detailed view of the dimerization interface in the transmembrane region as marked by box in (**a**). This dimerization interface is mainly mediated by the contacts between TM2 of one protomer and TM5 of the other protomer, which seems further secured by several ordered lipids: two phospholipids denoted as PL1 and PL2, three lipid alkyl chains denoted as L1-L3.

e, Cryo-EM densities of the ordered lipids (PL1, L1-3) identified in the dimer interface in (**d**). PL1 and PL2 are symmetry-related, so only PL1 is shown.

f, Close-up view of the boxed region (orange line) in (**d**) shows the residues on TM2 and TM5 involved in dimerization. This includes numerous hydrophobic residues on TM2 (V833, V836, W840, I844, L847, I851 and L852) and TM5 (Y1067, I1064, A1056, I1053, F1052, I1049, V1048, I1045 and I1044). Three

lipid alkyl chains may further secure this dimerization interface, including two in the extracellular membrane leaflet (L1 and L2) and one in the cytosolic leaflet (L3).

g, Close-up view of the boxed region (pink dashed line) in (**g**) shows that two ordered phospholipids (PL1 and PL2) are wrapped within the central membrane chamber at the dimerization interface. They are symmetry-counterparts located along the 2-fold symmetry axis. The membrane chamber is enclosed by TM2, TM5 from two protomers and is sealed by the interaction of I1087-I1087 from two protomers.

h, Close-up view of the boxed region (red line) in (**g**) shows the extensive interacting network to stabilize the two phospholipids, viewed at the orthogonal angle relative to (**g**). Lipid headgroup contacting R817 and lipid alky chains contacting numerous hydrophobic residues (F813, L839, W840, Y842, I844, L847, F850, V1048, F1052 and V1055). Only PL1 is shown since PL1 and PL2 are symmetry-related.

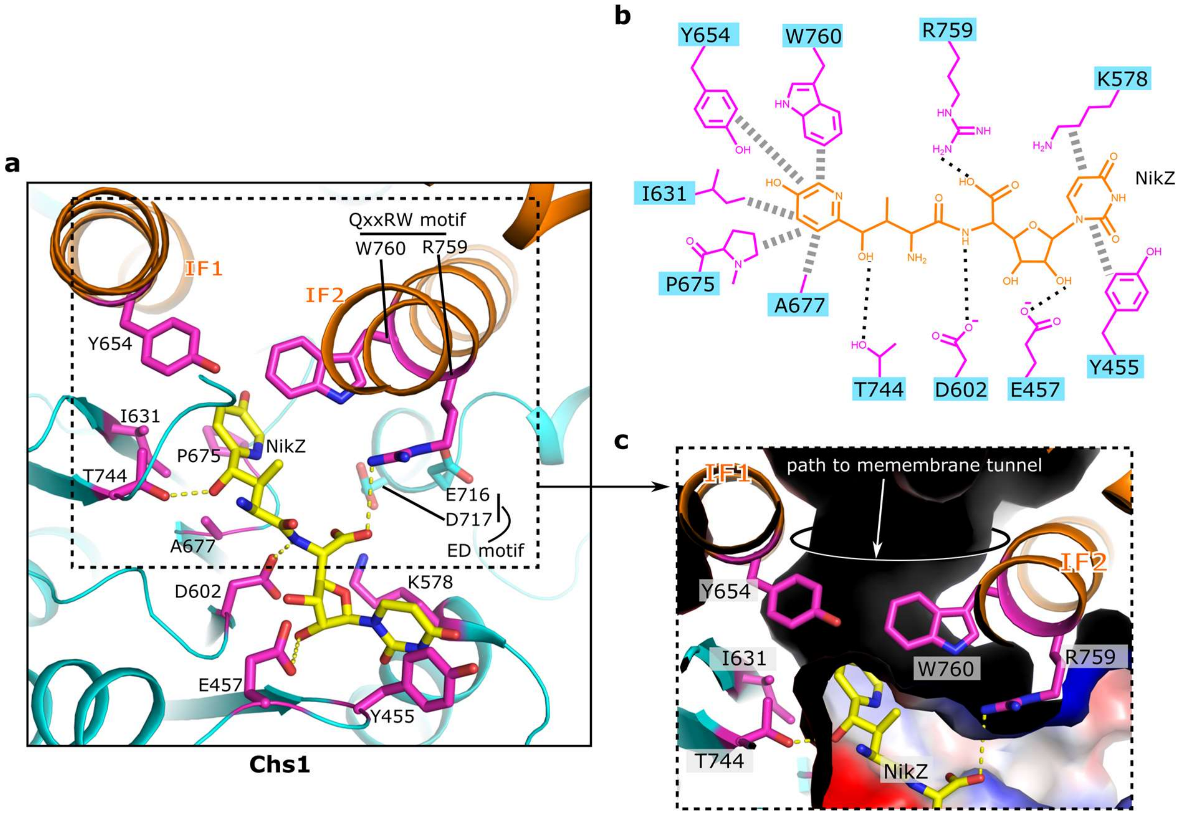

Supplementary Fig. S5. The binding of Nikkomycin Z in Chs1 active site.

a, Interaction between Chs1 and Nikkomycin Z. Residues mediating the interactions are shown as magenta sticks. Potential polar interactions are shown as yellow dashed lines. E716 and D717 (shown as cyan sticks) from the conserved 'ED motif', are close to but are not involved in the contacts of Nikkomycin Z.

b, Schematic of the interactions between Chs1 and Nikkomycin Z. Residues are colored as in (**a**), with hydrogen bonds indicated by black dashed lines and van der Waals contacts by thick grey dashed lines.

c, Clipped view of the cytosolic tunnel (in electrostatic surface representation) holding the Nikkomycin Z. Selective residues interacting with NikZ are shown as sticks.

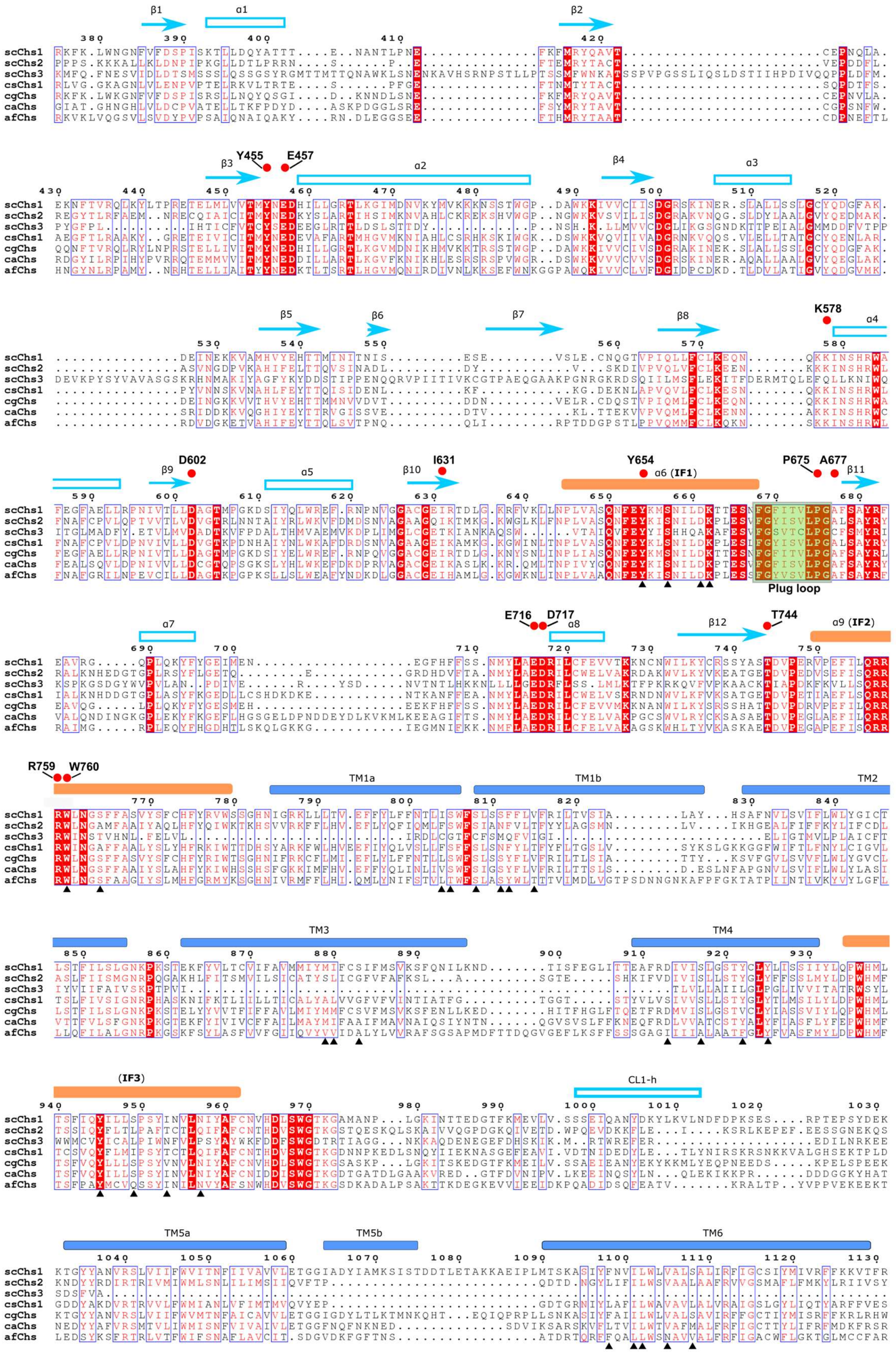
scChs1
scChs2
scChs3
csChs1
cgChs
caChs
afChs
β1
α1
β2
β3
Y455
E457
α2
β4
α3
β5
β6
β7
β8
K578
α4
β9
D602
α5
β10
I631
Y654
α6 (IF1)
P675
A677
β11
Plug loop
α7
E716
D717
α8
β12
T744
α9 (IF2)
R759
W760
TM1a
TM1b
TM2
TM3
TM4
(IF3)
CL1-h
TM5a
TM5b
TM6

Supplementary Fig. S6. Sequence analysis of chitin synthases from various pathogenic fungi and *Saccharomyces cerevisiae*.

Sequences are aligned using Clustal Omega server and are illustrated using ESPript server. Sequences are selected chitin synthases from *Saccharomyces cerevisiae* (sc), *Candida albicans* (cs), *Candida glabrata* (cg), *Candida auris* (ca) and *Aspergillus fumigatus* (af). Secondary structural elements of *S. cerevisiae* Chs1 are indicated above the alignment. Residues involved in NikZ binding or ligand binding/catalysis are highlighted with red dots. Residues lining the putative chitin translocation channel (as shown in **Supplementary Fig. S9d-e**) are marked by the black triangles under the alignment. Interfacial helices IF1-3 and functional motifs such as 'ED motif', 'QxxRW motif', 'plug loop' are labeled.

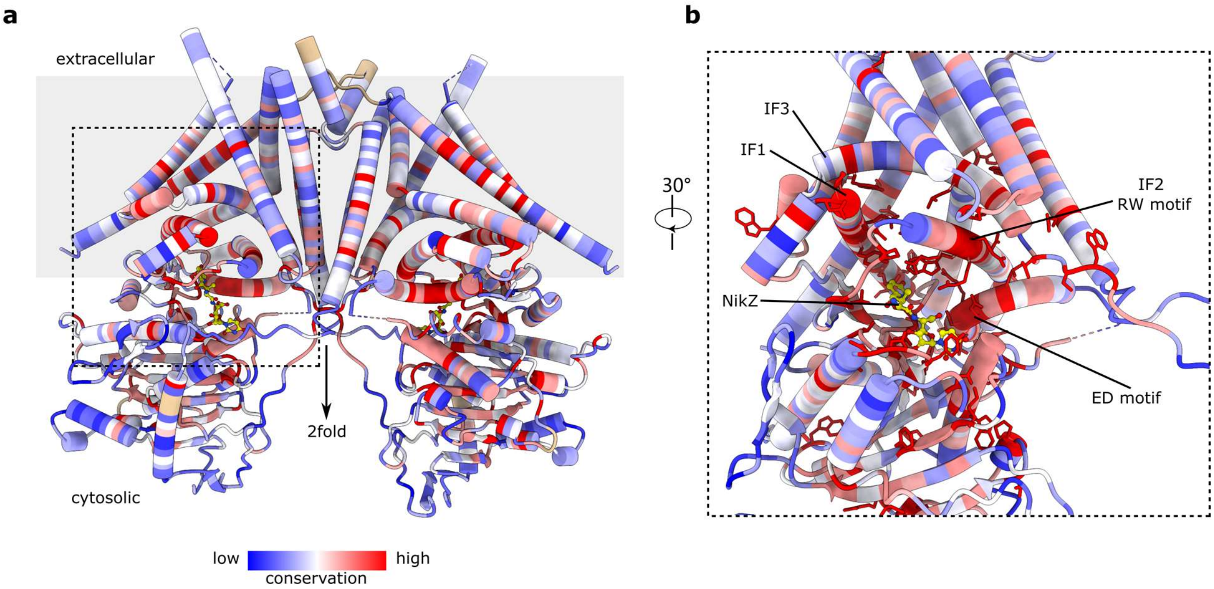

Supplementary Fig. S7. Sequence conservation of fungi chitin synthases illustrated on the structure of *S. cerevisiae* Chs1.

a, Sequence conservation (as shown in **Fig. S6**) is mapped onto the *S. cerevisiae* Chs1 dimer. The Chs1 dimer is colored according to the conservation level of each residue (red, high; blue, low), calculated by ChimeraX.

b, Close-up view of one Chs1 monomer shows the high conservation in its active site.

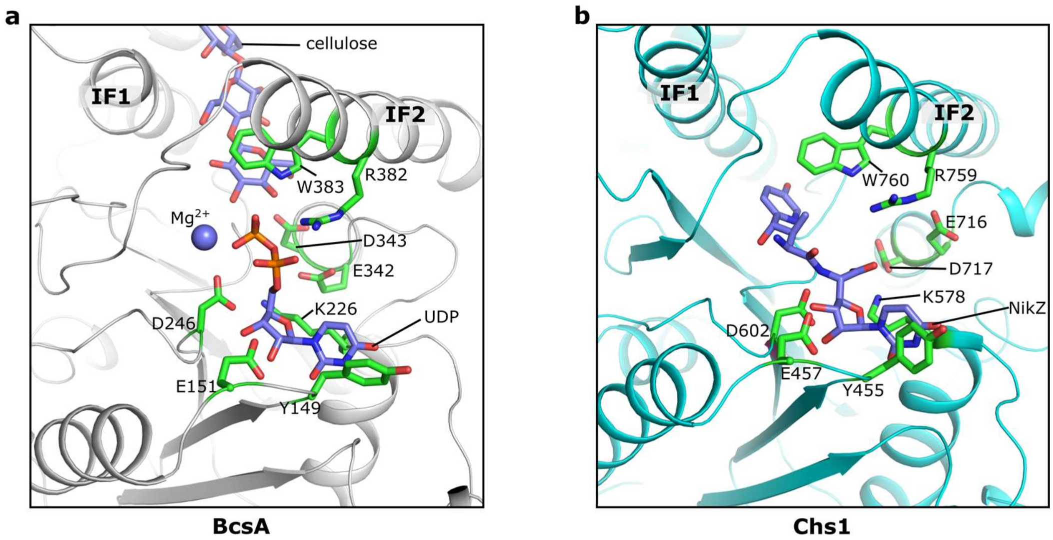

Supplementary Fig. S8. Structural comparison of the active sites between Chs1 and cellulose synthase BcsA. The GT catalytic domains of BcsA (panel **a**; PDB ID: 4p00) and Chs1 (panel **b**) are overlaid and shown separately as cartoon. Residues involved in catalysis or substrate binding are shown as sticks. The conserved residues are highlighted as green when using the active site of BcsA (**a**) as reference. The bound ligands are shown as blue sticks.

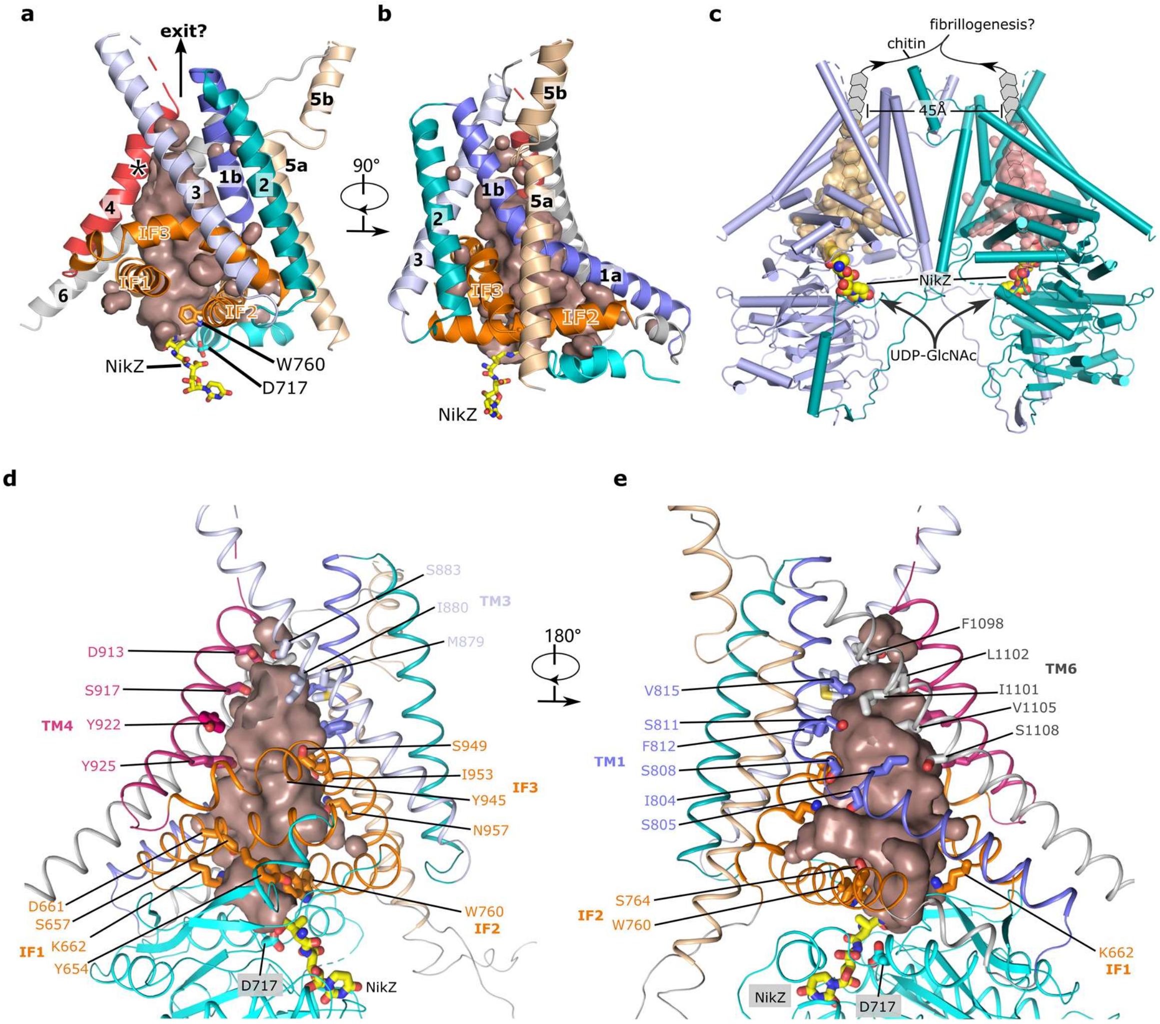

Supplementary Fig. S9. Membrane tunnel putative for chitin translocation.

a-b, The transmembrane domain encloses a membrane tunnel for chitin translocation. The membrane tunnel is shown in brown surface view and is displayed in two orthogonal views. The transmembrane helices and interface helices surrounding the tunnel are shown in cartoon representation.

c, Positions of two membrane tunnels (gold and red surface) in Chs1 dimer. Putative chitin translocation path is illustrated by the hexagon arrays.

d-e, The front and back view of the membrane tunnel (brown surface). Residues lining the tunnel are shown as sticks and colored according to their location.

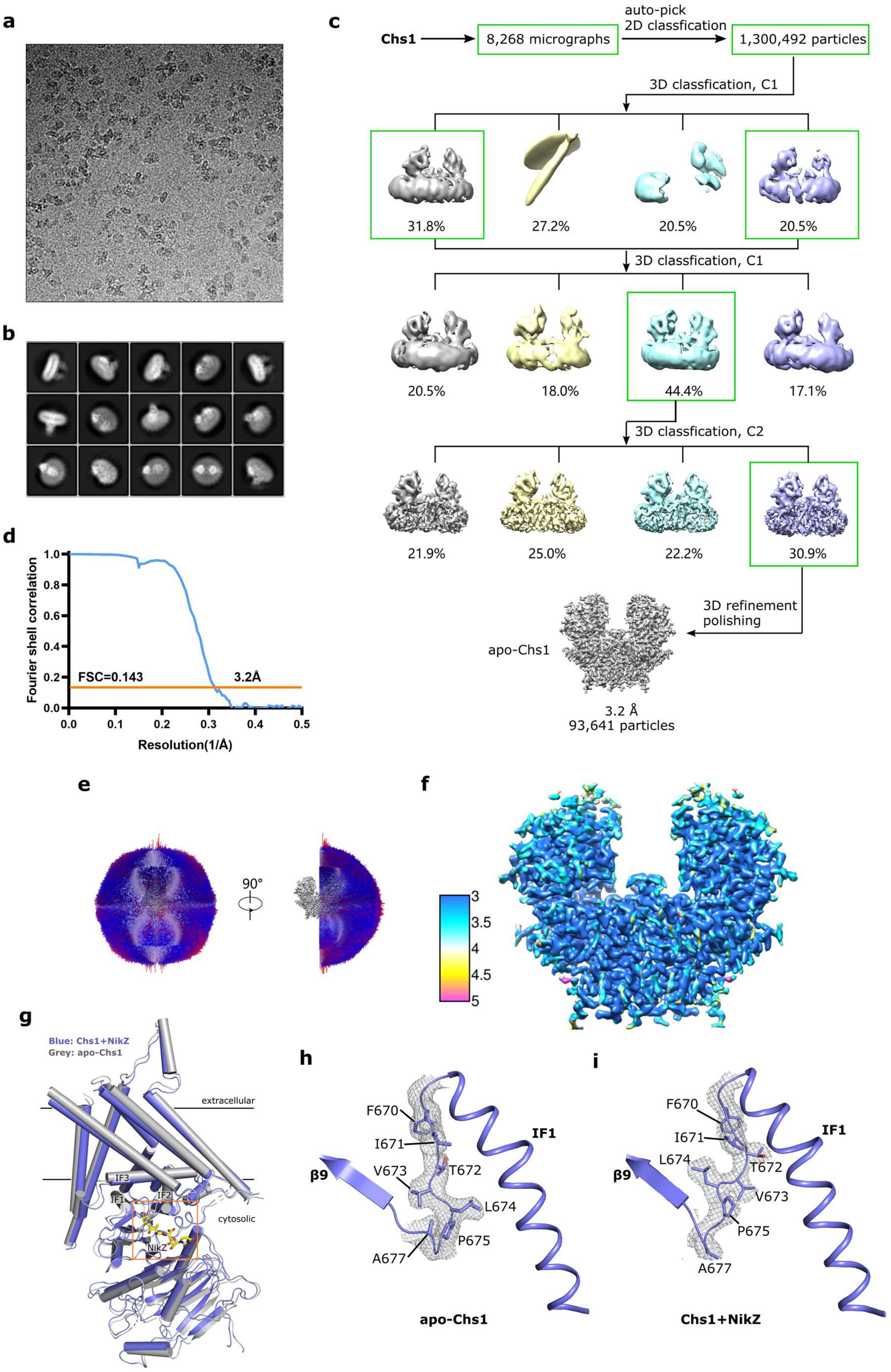
a
b
c
Chs1
8,268 micrographs
auto-pick
2D classfication
1,300,492 particles
3D classfication, C1
31.8%
27.2%
20.5%
20.5%
3D classfication, C1
20.5%
18.0%
44.4%
17.1%
3D classfication, C2
21.9%
25.0%
22.2%
30.9%
3D refinement
polishing
apo-Chs1
3.2 Å
93,641 particles
d
Fourier shell correlation
FSC=0.143
3.2Å
Resolution(1/Å)
e
90°
f
3
3.5
4
4.5
5
g
Blue: Chs1+NikZ
Grey: apo-Chs1
extracellular
cytosolic
IF3
IF1
IF2
NikZ
h
F670
I671
V673
T672
L674
P675
A677
β9
IF1
apo-Chs1
i
F670
I671
L674
T672
V673
P675
A677
β9
IF1
Chs1+NikZ

Supplementary Fig. S10. CryoEM analysis of apo state Chs1.

a, Representative cryo-EM micrograph of apo state Chs1.

b, Representative 2D class averages.

c, Flowchart for cryo-EM data processing.

d, Gold-standard Fourier shell correlation (FSC) curve.

e, Cutaway views of the angular distribution of all particles used in the final 3D reconstruction.

f, Local resolution distribution of the final cryo-EM map, calculated with ResMap.

g, Structural comparison between the apo-form Chs1 (grey cartoon) and Chs1 complexed with Nikkomycin Z (blue cartoon). The orange box marks the active site where Nikkomycin Z (yellow stick) binds and where the plug loops with altered conformations locate.

h-i, Comparison of the cryoEM densities at the plug loops between apo state Chs1 (**h**) and Chs1 bound with Nikkomycin Z (**i**), showing the conformational change of this loop.

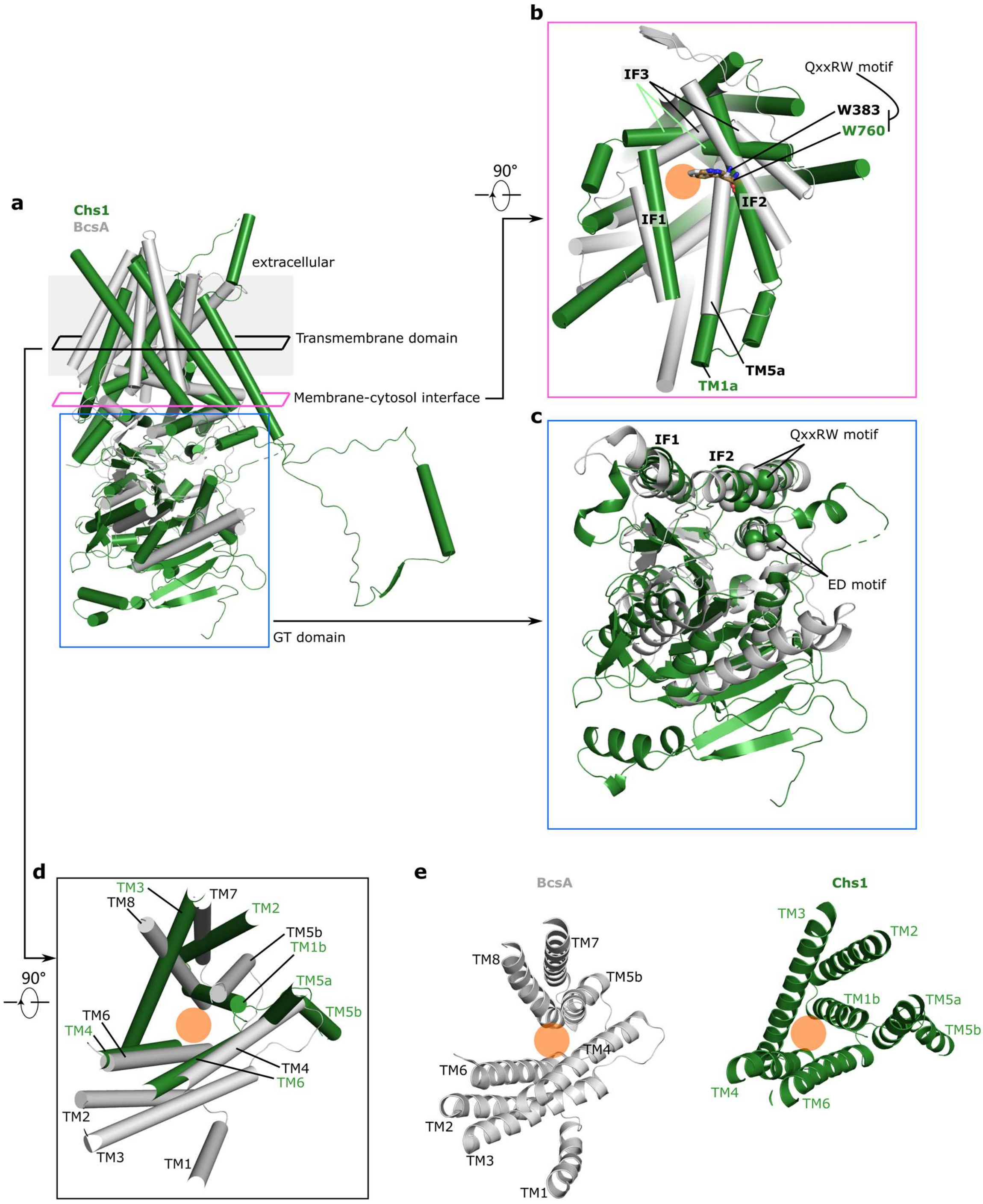

Supplementary Fig. S11. Structural comparison of two GT2 family members: chitin synthase Chs1 and cellulose synthase BcsA.

a, Structural superposition of Chs1 (green) and BcsA (grey, PDB ID: 4HG6).

b-d, Close-up views of selected regions as marked in the aligned structures in (**a**).

b, Structural elements located at the membrane-cytoplasm interface. Characteristic elements such as three interfacial helices IF1-IF3 and the broken TM1 are similarly organized to form the path connecting the membrane tunnel for chitin translocation, as indicated by the orange circle.
c, GT domains. Two interfacial helices IF1 and IF2, two characteristic motifs 'ED motif' and 'QxxRW motif' are indicated as labels.
d, Transmembrane domains. TM helices are denoted by numbers. Chs1 and BcsA contain membrane tunnels in their transmembrane domains (as marked by the orange circle), which are lined by differently organized TM helices.
e, The comparison of transmembrane domains in (**d**) is shown separately for BcsA (left) and Chs1 (right). The membrane tunnels for polysaccharide translocation are marked by the orange circles.

Supplementary Table S1. Cryo-EM data collection, refinement and validation statistics

	Chs1-NikZ	Chs1-apo
**Data collection and processing**		
Voltage (kV)	300	300
Electron exposure (e^{-}/Å^2)	50	50
Defocus range (μm)	-1 to -3	-1 to -3
Pixel size (Å)	1.08	0.92
Symmetry imposed	C2	C2
Initial particle images (no.)	820,166	1,300,492
Final particle images (no.)	188,337	93,641
Map resolution	2.9	3.2
**Refinement**		
Map sharpening *B* factor (Å^2)	-86.2	-93.0
Model composition		
Non-hydrogen atoms	12440	12270
Protein residues	1458	1458
Ligands	42 lipids; 2 NikZ	42 lipids
B factors (Å^2)		
Protein	37.0	60.3
Ligand	29.2	49.9
R.m.s. deviations		
Bond lengths (Å)	0.005	0.003
Bond angles (°)	0.754	0.669
Validation		
MolProbity score	1.8	1.5
Clashscore	7	4
Poor rotamers (%)	0.4	0.3
Ramachandran plot		
Favored (%)	94.5	95.5
Allowed (%)	5.3	4.2
Disallowed (%)	0.2	0.3
